# Estimating the prevalence of infectious diseases from under-reported age-dependent compulsorily notification databases

**DOI:** 10.1186/s12976-017-0069-2

**Published:** 2017-12-12

**Authors:** Marcos Amaku, Marcelo Nascimento Burattini, Eleazar Chaib, Francisco Antonio Bezerra Coutinho, David Greenhalgh, Luis Fernandez Lopez, Eduardo Massad

**Affiliations:** 1 LIM01-Hospital de Clínicas, Faculdade de Medicina Universidade de São Paulo, São Paulo, SP Brazil; 2 Hospital São Paulo, Escola Paulista de Medicina, Universidade Federal de São Paulo, São Paulo, SP Brazil; 3 Department of Mathematics and Statistics, The University of Strathclyde, Glasgow, Scotland UK; 4 Center for Internet Augmented Research & Assessment, Florida International University, Miami, FL USA; 5 London School of Hygiene and Tropical Medicine, London, UK

**Keywords:** Hepatitis C, Mathematical models, Notifications system incidence, Prevalence

## Abstract

**Background:**

National or local laws, norms or regulations (sometimes and in some countries) require medical providers to report notifiable diseases to public health authorities. Reporting, however, is almost always incomplete. This is due to a variety of reasons, ranging from not recognizing the diseased to failures in the technical or administrative steps leading to the final official register in the disease notification system. The reported fraction varies from 9 to 99% and is strongly associated with the disease being reported.

**Methods:**

In this paper we propose a method to approximately estimate the full prevalence (and any other variable or parameter related to transmission intensity) of infectious diseases. The model assumes incomplete notification of incidence and allows the estimation of the non-notified number of infections and it is illustrated by the case of hepatitis C in Brazil. The method has the advantage that it can be corrected iteratively by comparing its findings with empirical results.

**Results:**

The application of the model for the case of hepatitis C in Brazil resulted in a prevalence of notified cases that varied between 163,902 and 169,382 cases; a prevalence of non-notified cases that varied between 1,433,638 and 1,446,771; and a total prevalence of infections that varied between 1,597,540 and 1,616,153 cases.

**Conclusions:**

We conclude that the model proposed can be useful for estimation of the actual magnitude of endemic states of infectious diseases, particularly for those where the number of notified cases is only the tip of the iceberg. In addition, the method can be applied to other situations, such as the well-known underreported incidence of criminality (for example rape), among others.

## Background

Compulsory notifiable diseases (CNDs) are those diseases that should be compulsorily reported to Health Authorities as soon as suspected by the attending professional [1]. The notified cases then enter a database from which, among other things, it is possible to know the incidence (new cases per age, sex, risk factor, geographic location, etc., per period of time) of the disease. The availability of such information allows health authorities, in principle, to monitor and to plan controlling the disease, for example providing early warning of possible outbreaks [2].

In spite of international, national or local laws, norms or regulations requiring medical providers to report notifiable diseases to public health authorities, reporting is almost always incomplete [3–8]. This is due to a variety of reasons. First diseases may be asymptomatic. For example only around one in five dengue cases are symptomatic [9]. Second a case may be symptomatic but an individual may not seek healthcare due to mild or self-limiting symptoms or lack of knowledge about when to seek healthcare [4] or social stigma due to the nature of the disease, (for example sexually transmitted diseases). Even if an individual seeks healthcare a disease may not be notifiable, or if now notifiable may not have been notifiable in the past leading to incomplete notification records. A disease may also be misdiagnosed. Finally there may be failures in the technical or administrative steps leading to registration [10].

Rosenburg et al. [11] estimated that for every 100 persons infected with *Shigella*, 76 become symptomatic, 28 consulted a physician, nine submitted stool samples, seven had positive results, six were reported to the local health department and five were reported nationally to the Centers for Disease Control and Prevention. Thus they proposed a multiplication factor of 20 to estimate the number of *Shigella* infections based on national *Shigellosis* case reports.

Konowitz, Petrossian and Rose [10] investigated under-reporting of disease and knowledge of physicians of reporting requirements at two hospitals in New York City in 1982. They say that physicians may not know which diseases are reportable or the correct reporting procedures. The percentage of physicians who knew which diseases they had to report ranged from 37% for trachoma to 96% for syphilis. The results of Konowitz et al. suggest that a major factor in physician under-reporting is lack of knowledge of the reporting system.

Brabazon et al. [12] highlighted the extent of under-reporting of notifiable infectious disease hospitalisations in a health-board in Ireland, which was felt to be concerning for disease surveillance. Under-reporting was definitely demonstrated in 9 out of 22 notifiable diseases amounting to 572 cases (18% of missed cases). The most missed cases were viral meningitis, infectious mononucleosis, unspecified hepatitis C and acute encephalitis.

Keramou and Evans [5] performed a systematic review of completeness of infectious disease notification in the United Kingdom. Reporting completeness varied from 3 to 95% and was most strongly correlated with the disease being reported. Median reporting completeness was 73% for tuberculosis, 65% for meningococcus disease and 40% for other diseases. They conclude that reporting completeness remains suboptimal even for diseases that are under enhanced surveillance or were of significant public health importance.

A review by Doyle et al. [3], limited to published studies conducted in the United States between 1970 and 1999, quantitatively assessed infectious disease reporting completeness and found that reporting completeness varied from 9 to 99% and was strongly associated with the disease being reported. In another study [13] the mean reporting completeness for acquired immunodeficiency syndrome, sexually transmitted diseases, and tuberculosis as a group was significantly higher (79%) than for all other diseases combined (49%).

Schiffman et al. [14] investigated under-reporting of lyme and other tick-borne diseases in residents of a high incidence county, Minnesota, USA, in 2009. Of 444 illness events 352 (79%) were not reported. Of these 102 (29%) meet confirmed or probable surveillance case criteria including 91 (26%) confirmed lyme disease cases.

Serra et al. [8] developed a universal method to correct under-reporting of communicable diseases and applied it to incidence of hydatidosis in Chile, 1985-1994. According to this method the real rate of human hydatidosis in the period 1985-1994 was four times higher than the official notification in the given period.

Rowe and Cowie [6] used data linkage to improve the completeness of Aboriginal and Torres Strait Islander status in communicable disease notifications in Victoria, Australia. The burden of notifiable diseases in Torres Strait Islander Victorians could not be accurately estimated due to under-reporting of indigenous status. There were 12,488 cases of hepatitis B, hepatitis C (HCV) and gonococcal infection in Victoria in 2009-2010 with indigenous status missing in 61.6, 67.8 and 33.1% of those conditions, respectively. They used data linkage to improve completeness of indigenous status in people notified with viral hepatitis and gonococcal infection.

Of particular concern are those chronic, mainly asymptomatic, infectious diseases that allow infected individuals to live for years or even decades without being recognised as such. These diseases can represent a heavy burden to the affected populations and pose significant risk to the international community. Perhaps the most dramatic examples of the latter include human immunodeficiency (HIV) and HCV viruses pandemics. In fact, these two infections have been labeled by WHO as the epidemics of the XXth and XXIth centuries, respectively [7, 15].

One critical consequence of under-notification of such diseases is the fact that their prevalence estimates are frequently way under-estimated, leading to miscalculation of their actual burden and making control efforts suboptimal [4].

HCV is a disease with a long period between infection and symptoms developing. Because infected people are mainly asymptomatic and risk behaviour may have occurred a long time ago individuals often do not consult health professionals to discuss potential disease infection. As in general a large high risk group is people who share injection equipment and other injection paraphernalia, for example cookers, filters and spoons, and drug injection is an illegal activity, which often does not meet with social approval, light to moderate injectors, or past injectors who do not currently inject, may not disclose their risky behaviour to their health provider. Being unaware of the risk behaviour the health provider is unable to recommend HCV screening. Also HCV is extremely easy to catch via injecting. Past injectors who no longer inject may not perceive themselves to be at risk.

In a previous paper [16] we assumed that the infection (HCV) was in steady-state. Then we proposed two methods to give a first rough estimate of the actual number of HCV infected individuals (prevalence) taking into account the yearly notification rate of newly reported infections (incidence of notification) and the size of the Liver Transplantation Waiting List (LTWL) of patients with liver failure due to chronic HCV infection [17]. Both approaches, when applied to the Brazilian HCV situation converged to the same results, that is, the methods proposed reproduce both the prevalence of reported cases and the LTWL with reasonable accuracy. In that paper we show how to calculate the prevalence of people living with HCV in Brazil, which resulted in a value up to 8 times higher than the official reported number of cases [16].

In both [16] and this paper the under-reporting mechanism is included in the model by dividing the infected individuals into two categories: notified and non-notified. Newly infected individuals enter the non-notified class and leave it either through death, recovery or notification. If they are notified they immediately enter the notified infected class.

The present paper is an improvement of those techniques because, unlike in the previous paper mentioned above, now we do not assume steady state. Unfortunately, given the short period of time with data available (hepatitis notification became compulsory in Brazil only in 1999 [18], it cannot give more precise information on HCV prevalence than the one already provided by our previous study, but it illustrates the techniques that allow the prevalence estimation based on age and time of previous notifications, and that can be applied to any notifiable disease.

This paper is organised as follows: First we describe a continuous model, that is a model where the variables are continuous functions of age and time. Next we describe a discrete model, in which the variables are discrete functions of age and time. In the following section we discuss application to HCV. Then we turn to our estimation method applied to the size of the Liver Transplantation Waiting List in Brazil. The next section gives our numerical results. Discussion and conclusions close the paper.

## Methods

### Continuous time and age model

Assume we have an SIR (Susceptible-Infected-Removed) type infection and let *S*(*a*, *t*)*da*, *I*(*a*, *t*)*da* and *R*(*a*, *t*)*da* be the number of individuals with age between *a* and *a* + *da* at time *t* that are susceptible, infected and removed (or recovered), respectively. In addition, as mentioned in the Background section, public health authorities demand that some diseases be compulsorily notifiable, that is they publish the number of diagnosed individuals per time unit for each age interval (incidence) in public databases. Therefore, we can divide the prevalence of infected individuals into two classes: notified individuals, denoted *I* ^*N*^(*a*, *t*)*da*, and non-notified individuals, denoted *I* ^*NN*^(*a*, *t*)*da*.

Let *λ*(*a*, *t*)be the so-called age and time-dependent force-of-infection (incidence density). Then:

1 $$ \lambda \left(a,t\right)S\left(a,t\right) dadt $$

is the number of susceptible individuals who get the infection when aged between *a* and *a* + *da* during the time interval *dt*. Standard arguments allow us to write the following system of partial differential equations, known as Trucco-Von Foester equations in the literature [19]:

2 $$ {\displaystyle \begin{array}{l}\frac{\partial S\left(a,t\right)}{\partial t}+\frac{\partial S\left(a,t\right)}{\partial a}=-\lambda \left(a,t\right)S\left(a,t\right)-\mu \left(a,t\right)S\left(a,t\right),\\ {}\frac{\partial {I}^{NN}\left(a,t\right)}{\partial t}+\frac{\partial {I}^{NN}\left(a,t\right)}{\partial a}=\lambda \left(a,t\right)S\left(a,t\right)\\ {}\kern1.50em -\left(\mu \left(a,t\right)+{\alpha}^{NN}\left(a,t\right)+{\gamma}^{NN}\left(a,t\right)\right){I}^{NN}\left(a,t\right)-\kappa \left(a,t\right){I}^{NN}\left(a,t\right),\\ {}\frac{\partial {I}^N\left(a,t\right)}{\partial t}+\frac{\partial {I}^N\left(a,t\right)}{\partial a}=\kappa \left(a,t\right){I}^{NN}\left(a,t\right)-\left(\mu \left(a,t\right)+{\alpha}^N\left(a,t\right)+{\gamma}^N\left(a,t\right)\right){I}^N\left(a,t\right),\\ {}\frac{\partial R\left(a,t\right)}{\partial t}+\frac{\partial R\left(a,t\right)}{\partial a}={\gamma}^{NN}\left(a,t\right){I}^{NN}\left(a,t\right)+{\gamma}^N\left(a,t\right){I}^N\left(a,t\right)-\mu \left(a,t\right)R\left(a,t\right),\end{array}} $$

where the meaning of the parameters is described in Table 1.

**Table 1:** Parameters used in model (2)

Parameter	Meaning	Values used in the numerical simulation
*λ*(*a*, *t*)	Force of Infection	Calculated
*μ*(*a*, *t*)	Natural Mortality Rate	0.0133 year^-1a^
*α* ^*NN*^(*a*, *t*)	Disease-induced Mortality Rate for non-notified individuals	^b^
*α* ^*N*^(*a*, *t*)	Disease-induced Mortality Rate for notified individuals	^b^
*γ* ^*NN*^(*a*, *t*)	Recovery Rate for non-notified individuals	Assumed negligible
*γ* ^*N*^(*a*, *t*)	Recovery Rate for notified individuals	Assumed negligible
*κ*(*a*, *t*)	Notification Rate	0.0125 year^-1^ [16]

^a^From demographic data of Brazil

^b^Constructed as equal to 0*.*15/{1 + exp.(−0*.*1(*a* − 57*.*31))} year^−1^ as in [16]

In Table 1, we neglected the value of the recovery rates in the numerical simulations because we assumed that HCV infection is very long-lasting. These parameters, however, were included in the model for the sake of completeness.

The notification rate *κ*(*a*, *t*) is one of the most important parameters in the model. This represents the rate at which those non-notified individuals of age *a* are reported to health authorities and notified. This has two components, first the rate of an infected person being recognised and secondly the rate of being reported. So if *κ*(*a*, *t*) is small then there will be a large number of non-notified infected individuals hidden from the system, whereas if *κ*(*a*, *t*) is large then most infected individuals will be notified and the records will accurately reflect the number infected in the population.

The solution of system (2) can be obtained with the method of characteristics [19]. However, for our purposes, it is better to solve the equation by following a cohort, as described in [20].

The solution of the equation for susceptible individuals is:

3 $$ S\left(a,{t}_0+a\right)=S\left(0,{t}_0\right)\exp \left(-{\int}_0^a\left[\lambda \left(s,{t}_0+s\right)+\mu \left(s,{t}_0+s\right)\right] ds\right). $$

There are a small number of maternal-infant HCV infections [21]. It would be possible to include these in the theoretical model. However data for age zero is not used in the calculations because it is unreliable. So to include maternal-infant HCV infections would make the model more complicated but not change the numerical results. So we ignore these maternal-infant HCV infections.

The solution for the equation for infected individuals is:

4 $$ {\displaystyle \begin{array}{l}{I}^{NN}\left(a,{t}_0+a\right)={\int}_0^a\lambda \left(s,{t}_0+s\right)S\left(s,{t}_0+s\right)\\ {}\exp \left(-{\int}_s^a\left[\mu \left(x,{t}_0+x\right)+{\gamma}^{NN}\left(x,{t}_0+x\right)+{\alpha}^{NN}\left(x,{t}_0+x\right)+\kappa \left(x,{t}_0+x\right)\right] dx\right)\  ds,\end{array}} $$

5 $$ {\displaystyle \begin{array}{l}{I}^N\left(a,{t}_0+a\right)={\int}_0^a\kappa \left(s,{t}_0+s\right){I}^{NN}\left(s,{t}_0+s\right)\\ {}\kern2.25em \exp \left(-{\int}_s^a\left[\mu \left(x,{t}_0+x\right)+{\gamma}^N\left(x,{t}_0+x\right)+{\alpha}^N\left(x,{t}_0+x\right)\right] dx\right)\  ds.\end{array}} $$

Finally, the equation for the removed individuals is given by:

6 $$ {\displaystyle \begin{array}{r}R\left(a,{t}_0+a\right)={\int}_0^a\left({\gamma}^{NN}\left(s,{t}_0+s\right){I}^{NN}\left(s,{t}_0+s\right)+{\gamma}^N\left(s,{t}_0+s\right){I}^N\left(s,{t}_0+s\right)\right)\\ {}\kern0.5em \exp \left(-{\int}_s^a\left[\mu \left(x,{t}_0+x\right)\right] dx\right)\  ds.\hfill \end{array}} $$

Assuming steady state, the system (1) was solved by Amaku et al. [16] to calculate the prevalence of HCV in Brazil. The work that follows is an extension of the methods described there and its results are in accordance with the previous results for the cases where real data are available.

### Discrete time and age model

In real life epidemics notification is discrete with the time and age units expressed in weeks, months or years. Hence, in order to apply the model to a real public health problem we discretised model (2), with time and age unit expressed in years. This discretisation has to be done carefully to use the maximum advantage of the data available.

### Calculating the prevalence *I*^NN*^{*A,i*} and *I*^N*^{*A,i*}

To avoid potential confusion between similar variables in the discrete and continuous models we adopt the convention that discrete variables have a ‘*’ superscript after the variable and their arguments are in curly parentheses, {}, whereas continuous variables do not have a ‘*’ superscript after the variable and their arguments are in round parentheses ().

From the SINAN database we can calculate SINAN*{*A*,*i*} where *A* is an integer number and *i* represents a calendar year, which represents the number of infected individuals notified to SINAN in the calendar year *i*, who at the end of calendar year *i* have age *A* years (in other words at the end of calendar year *i* their exact age *a* is in the time interval [*A*,*A* + 1)).

Because we want the variables in the discrete model to relate to the SINAN data we similarly define ${I}^{NN^{\ast }}\left\{A,i\right\}$ and ${I}^{N^{\ast }}\left\{A,i\right\}$ to denote respectively the number of non-notified infected and notified infected individuals at time the end of calendar year *i*, whose age at that time is *A* years (so their exact age lies in [*A*,*A* + 1)). Given parametric functions such as *κ*(*a*, *t*) and *ϕ* ^*NN*^(*a*, *t*) in the continuous model, in the corresponding discrete model these are assumed to be discrete functions *κ*_*d*_(*a*, *t*) = *κ*_*A*, *i*_ and ${\phi}_d^{NN}\left(a,t\right)={\phi}_{A,i}^{NN}$ for (*a*, *t*) ∈ *R* = {*a* ∈ [*A*, *A* + 1) and *t* ∈ (*t*_*i*_ − 1, *t*_*i*_]}. Here *t*_*i*_ denotes the end of calendar year *i*, and *κ*_*A*, *i*_ and ${\phi}_{A,i}^{NN}$ are respectively the average values of *κ*(*a*, *t*) and *ϕ* ^*NN*^(*a*, *t*) over the region *R*.

The discretised versions of Eqs. (4) and (5) are given by Eqs. (7) and (8) below, which are approximations as explained in the Appendix.

7 $$ {\displaystyle \begin{array}{c}{I}^{NN\ast}\left\{A,i\right\}={I}^{NN\ast}\left\{A-1,i-1\right\}\exp \left[-\frac{1}{2}\left({\kappa}_{A-1,i}+{\kappa}_{A,i}+{\phi}_{A-1,i}^{NN}+{\phi}_{A,i}^{NN}\right)\right]\\ {}+ INC\left\{A,i\right\},\end{array}} $$

where for *A* = 0, *I* ^*NN*∗^{*A* − 1, *i* − 1} = 0. *INC*{*A*, *i*} is the new HCV cases occurring between times *t*_*i*_-1 and *t*_*i*_ that are still alive, infectious and non-notified at time *t*_*i*_ in the year cohort born between times *t*_*i*_ *-A*-1 and *t*_*i*_-*A*. Here (using the continuous model notation)

$$ {\phi}^{NN}\left(a,t\right)=\mu \left(a,t\right)+{\gamma}^{NN}\left(a,t\right)+{\alpha}^{NN}\left(a,t\right). $$

In Eq. (7), the term

$$ \exp \left[-\frac{1}{2}\left({\kappa}_{A-1,i}+{\kappa}_{A,i}+{\phi}_{A-1,i}^{NN}+{\phi}_{A,i}^{NN}\right)\right] $$

means the probability of not being removed from the non-notified class of individuals, either by natural death, disease-induced death, recovery or notification in the interval (*t*_*i*_-1,*t*_*i*_]. Equation (7) is very important because, as shown later in the paper, it allows the calculation of the true incidence from empirical data (see Eq. (12) below).

Recurrence Eq. (7) can be solved by well-known methods and the prevalence of notified and non-notified individuals can be estimated (see Eqs. (13) and (14) below).

Similarly, we can write:

8 $$ {\displaystyle \begin{array}{l}{I}^{N\ast}\left\{A,i\right\}={I}^{N\ast}\left\{A-1,i-1\right\}\exp \left[-\frac{1}{2}\left({\phi}_{A-1,i}^N+{\phi}_{A,i}^N\right)\right]\\ {}\kern2.50em +{\int}_A^{A+1} NOTIFICATION\left(a,\left({t}_i-1,{t}_i\right]\right) da,\end{array}} $$

where (again using the continuous model notation) *ϕ* ^*N*^(*a*, *t*) = *μ*(*a*, *t*) + *γ* ^*N*^(*a*, *t*) + *α* ^*N*^(*a*, *t*). The last term represents the notifications of HCV between times *t*_*i*_-1 and *t*_*i*_ of individuals in the year cohort born in *t*_*i*_-*A*-1 to *t*_*i*_-*A* who are still in the notified class at time *t*_*i*_, i.e.

9 $$ {\displaystyle \begin{array}{c}{\int}_A^{A+1}{\int}_0^1{\kappa}_d\left(a-1+x,{t}_i-1+x\right){I}^{NN}\left(a-1+x,{t}_i-1+x\right)\kern9.75em \\ {}\kern8.25em \exp \left[-{\int}_x^1{\phi}_d^N\left(a-1+z,{t}_1-1+z\right)\right] dx\kern0.24em da,\\ {}\approx {\kappa}_d\left(A+\frac{1}{2},{t}_i\right){I}^{NN}\left(A+\frac{1}{2},{t}_i\right).\end{array}} $$

This is because both integration intervals are of length one, hence to first order we can approximate the integrand by its value at any specific point in the integrated area. So we choose $a=A+\frac{1}{2}$, *x =* 1. Now note that

(i) ${\kappa}_d\left(A+\frac{1}{2},{t}_i\right)=$ *κ*_*A*, *i*_, as in the discrete model *κ*_*d*_(*a*, *t*) = *κ*_*A*, *i*_ over the region

*R* = {*a* ∈ [*A*, *A* + 1) and *t* ∈ (*t*_*i*_ − 1, *t*_*i*_]},

and 

(ii) $$ {I}^{NN^{\ast }}\left\{A,i\right\}\approx {I}^{NN}\left(A+\frac{1}{2},{t}_i\right), $$

as explained in the Appendix (Eq. (A5)). Hence the last term in (8) is

10 $$ \kern4em {\int}_A^{A+1} NOTIFICATION\left(a,\left({t}_i-1,{t}_i\right]\right) da\approx {\kappa}_{A,i}\ {I}^{NN^{\ast }}\left\{A,i\right\}. $$

In the next section, we are going to show how to solve Eqs. (7) and (8) using the notified cases in a particular setting, namely HCV in Brazil. Using the notified incidences and good guesses for the mortality rates we can calculate any desired properties of the infected population. In the next section we calculate the prevalence of the disease. The calculation presented applies to any notifiable infectious disease.

### Example of application: Hepatitis C

**Fig. 1:**
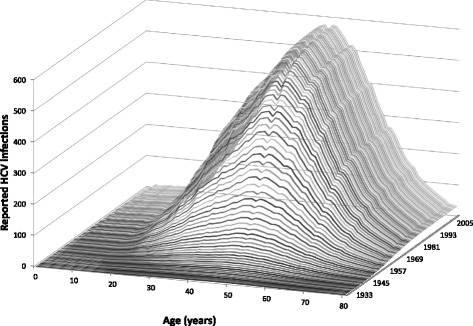
Time and Age variation of the reported number of HCV infections in Brazil, artificially constructed by extrapolating backwards until 1932

In this section we exemplify the above theory by calculating the prevalence of HCV, a flaviviral infection that afflicts close to 3% of the world population [22], in Brazil. As mentioned in the Introduction, the great majority of infections with HCV, however, are not easily identified and, therefore, frequently non-notified. Our data were taken from the National Reportable Disease Information System "Sistema de Informação de Agravos de Notificação" (SINAN) of the Brazilian Health Ministry [23]. SINAN is publicly available through the internet and used by the World Health Organisation [24]. It is used throughout Brazil, in all health institutions whether public or private. All Brazilians diagnosed with HCV are reported to SINAN. The database includes symptomatic patients who report to a doctor, also symptomatic individuals picked up through screening for blood banks or other means. The individuals are diagnosed and then the diagnosis is confirmed via an HCV antibody test. Figure 1 shows the time and age variation in the reported number of HCV cases in Brazil.

In fact, the actual number of reported HCV infections is available only from 2000 onward. As we know from previous studies [25], HCV was introduced in Brazil in the later 1950s. We therefore constructed the number of reported with a sigmoidal decay backwards until 1932, as argued below. We used this artifice only to illustrate the model and these figures have little epidemiological significance, as argued below. We shall return to this point in the results section, where we explain this procedure in more detail.

### Estimating the total number of HCV infected individuals in Brazil

Recall that *SINAN* ^*^{*A*,*i*} is the number of individuals aged *A* to *A* + 1 at time *t*_*i*_ who were notified to SINAN in the current year *i*, (*t*_*i*_-1,*t*_*i*_]. Now

11 $$ {SINAN}^{\ast}\left\{A,i\right\}\approx {\kappa}_{A,i}{I}^{NN\ast}\left(A,i\right). $$

This approximation is obtained by using Eq. (10) as

$$ {SINAN}^{\ast}\left\{A,i\right\}={\int}_A^{A+1} NOTIFICATION\left(a,\left({t}_i-1,{t}_i\right]\right) da.\kern0.5em $$

As HCV infection is determined by taking an antibody test it is not possible to distinguish between individuals protected by maternal antibodies from HCV infected individuals. Hence we do not use the data for *A* = 0 as it is unreliable, instead we take *SINAN* ^*^{*0,i*} = 0, for all *i*. Because only a very small number of individuals of age 0 are infected this does not cause significant error in the estimation.

From (7) and (11) we can write down the fundamental equation for estimating the incidence, for *A* ≥ 0:

12 $$ {\displaystyle \begin{array}{c} INC\left\{A,i\right\}=\frac{SINAN^{\ast}\left\{A,i\right\}}{\kappa_{A,i}}\\ {}-\frac{SINAN^{\ast}\left\{A-1,i-1\right\}}{\kappa_{A-1,i-1}}\exp \left\{-\frac{1}{2}\left({\kappa}_{A-1,i}+{\kappa}_{A,i}+{\phi}_{A-1,i}^{NN}+{\phi}_{A,i}^{NN}\right)\right\},\end{array}} $$

where SINAN*{0,*i*} and SINAN*{−1,*i*} are interpreted as zero for all *i*.

Note that, as observed in Eq. (12), the method consists of subtracting consecutive values of a diagonal of a matrix containing age in lines and time in columns. In some instances, however, it may happen that for certain ages and years the calculated incidence is negative. Our interpretation is that, for that particular age and time, the notified incidence was zero. When this happened in the actual calculation we assigned the value zero to the notification incidence.

Therefore, *I* ^NN*^{*A*,*i*} can be calculated for each age and time reported as

13 $$ {\displaystyle \begin{array}{c}{I}^{NN\ast}\left\{A,i\right\}=\sum \limits_{j=0}^A INC\left\{A-j,i-j\right\}\\ {}\ \exp \left\{-\frac{1}{2}\sum \limits_{p=0}^{j-1}\left({\kappa}_{A-1-p,i-p}+{\kappa}_{A-p,i-p}+{\phi}_{A-1-p,i-p}^{NN}+{\phi}_{A-p,i-p}^{NN}\right)\right\}.\end{array}} $$

Similarly, for *I* ^N*^{*A*,*i*}, we have:

14 $$ \kern0.5em {I}^{N\ast}\left\{A,i\right\}=\sum \limits_{j=0}^A{SINAN}^{\ast}\left\{A-j,i-j\right\}\exp \left\{-\frac{1}{2}\sum \limits_{p=0}^{j-1}\left({\phi}_{A-1-p,i-p}^N+{\phi}_{A-p,i-p}^N\right)\right\}. $$

**Fig. 2:**
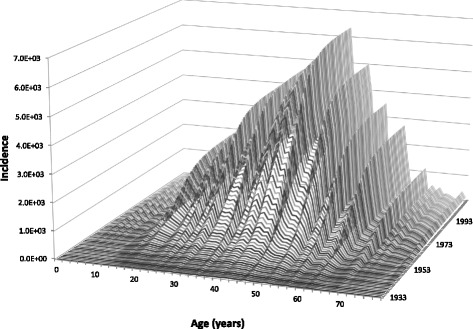
Calculation of *INC*{*A*, *i*} from the SINAN data as shown in Fig. 1

Figure 2 shows the calculation of *INC*{*A*, *i*} using Eq. (12) with the SINAN data as shown in Fig. 1.

### The size of the liver transplantation waiting list in Brazil

It is known that a fraction of those individuals infected with HCV evolve to liver failure after many years of infection [26]. Let us denote those individuals diagnosed with liver failure of whose age in whole years is *A* at the end of calendar year *i*, time *t*_*i*_ as *LF*{*A*, *i*}. These individuals have been necessarily diagnosed with HCV and, therefore, are a fraction of the notified infected individuals *I* ^N*^{*A*,*i*}. It is assumed that individuals develop liver failure after a minimum time interval *τ*_*min*_, say 10 years. From Eq. (8) for *I* ^N*^{*A*,*i*} we obtain the equation for *LF*{*A*, *i*}:

15 $$ LF\left\{A,i\right\}=\sum \limits_{\tau ={\tau}_{min}}^A{\eta}_{A-\tau }{I}^{N\ast}\left\{A-\tau, i-\tau \right\}\exp \left\{-\frac{1}{2}\left[\sum \limits_{p=0}^{\tau -1}\left({\varphi}_{A-1-p,i-p}^N+{\varphi}_{A-p,i-p}^N\right)\right]\right\}, $$

where *η*_*A* − *τ*_ is a discretised function that decreases from *τ* = *τ*_*min*_ up until *τ* = *A*, representing the rate at which infected (and notified) individuals of age *A-τ* develop liver failure.

We know that liver damage (whether due to HCV or some other cause) is a progressive disease [27, 28] so the longer that an individual has been infected the more liver damage they will have sustained and the greater the chance of liver failure. Given a group of individuals currently all of age *A* those that have been in the database longer are also more likely to have been infected for longer. Hence, *η*_*A* − *τ*_, the liver failure rate of those of current age *A* who were notified to the database *τ* years ago should increase with *τ*. Since early symptoms of liver disease precede complete failure it is reasonable to assume that there is a minimum gap between notification and liver failure.

Summing up over all ages we obtain the size of *LF*{*i*}, which is the total number of individuals with liver failure at time *t*_*i*_:

16 $$ LF\left\{i\right\}=\sum \limits_{A_{min}}^{A_{max}}\sum \limits_{\tau ={\tau}_{min}}^A{\eta}_{A-\tau }{I}^{N\ast}\left\{A-\tau, i-\tau \right\}\mathit{\exp}\left\{-\frac{1}{2}\left[\sum \limits_{p=0}^{\tau -1}\left({\varphi}_{A-1-p,i-p}^N+{\varphi}_{A-p,i-p}^N\right)\right]\right\}, $$

where *A*_min_ and *A*_max_ are minimum and maximum ages. Apart from those individuals who are transplanted (see below) *LF*{*i*} corresponds to the Liver Transplantation Waiting List (LTWL).

Let us now rewrite Eq. (16) considering transplantation. Let *ψ*(*a*, *t*) be the transplantation rate of individuals of aged *a* ∈ [*A*, *A* + 1) in calendar year *t* ∈ (*t*_*i*_ − 1, *t*_*i*_]. Then, Eq. (16) becomes

17 $$ {\displaystyle \begin{array}{c} LWTL\left\{i\right\}=\sum \limits_{A_{min}}^{A_{max}}\sum \limits_{\tau ={\tau}_{min}}^A{\eta}_{A-\tau }{I}^{N\ast}\left\{A-\tau, i-\tau \right\}\ \\ {}\ \exp \left\{-\frac{1}{2}\left[\sum \limits_{p=0}^{\tau -1}\left({\varphi}_{A-1-p,i-p}^N+{\varphi}_{A-p,i-p}^N+{\psi}_{A-1-p,i-p}^N+{\psi}_{A-p,i-p}^N\right)\right]\right\}.\end{array}} $$

The number of transplants in calendar year *i* is then given by *TR*{*i*} where

18 $$ {\displaystyle \begin{array}{c} TR\left\{i\right\}=\sum \limits_{A_{min}}^{A_{max}}\sum \limits_{\tau ={\tau}_{min}}^A{\psi}_{A,i}{\eta}_{A-\tau }{I}^{N\ast}\left\{A-\tau, i-\tau \right\}\kern0.5em \\ {}\kern0.75em \exp \left\{-\frac{1}{2}\left[\sum \limits_{p=0}^{\tau -1}\left({\varphi}_{A-1-p,i-p}^N+{\varphi}_{A-p,i-p}^N+{\psi}_{A-1-p,i-p}^N+{\psi}_{A-p,i-p}^N\right)\right]\right\}.\end{array}} $$

We take for *ψ*_*A*, *i*_ a suitably truncated bell-shaped discrete function [26] with a maximum at 45 years of age for all *i*.

## Results

One of our objectives is to calculate Eqs. (13) and (14) in order to obtain the estimated prevalence of notified and non-notified HCV infections which sum up to total prevalence. Unfortunately, the data available are restricted to the period between 2000 and 2012. In order to simulate a longer history of HCV infection in Brazil, we artificially constructed such a previous history by extrapolating backwards. First, we averaged the notified cases in the period between 2000 and 2012. Then, we fitted a sigmoidal-shaped curve representing the notified cases back for the period between 1932 and 2000. We did that for all ages such that the age distribution of notified cases was assumed fixed for all the extrapolated periods. We are well aware that HCV was probably introduced in Brazil in the 1950’s and, therefore, this calculation is only an exercise to illustrate the method.

**Fig. 3:**
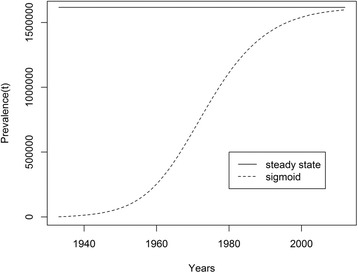
Comparison of the total prevalence calculated according to Amaku et al. [16] (continuous line) and assuming the notification as a sigmoidal extrapolation (dotted line)

In a previous paper [16], this extrapolation was done differently. We assumed the disease to be in steady state until 1932. The results of this previous calculation are therefore different from the ones presented in this paper. We shall elaborate on this later. To begin with, Fig. 3 shows a preliminary result on this direction. The continuous line is the total prevalence extrapolating the data as if in steady state [16]. The sigmoid dotted line is the total prevalence calculated assuming the artificially constructed notification as explained above.

**Fig. 4:**
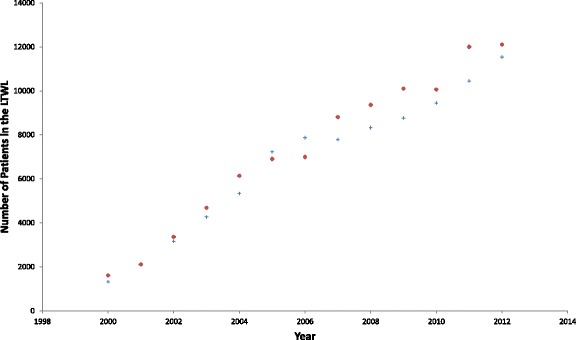
Comparison between the empirical data on the size of the LTWL (crosses) as in Chaib et al. [17] and the result of the application of Eq. (17) (dots)

Results of the numerical calculations are summarised in Table 2. In it we compare the prevalence in 2012 of HCV infected individuals who have been reported to SINAN until 2012 with the outcomes of the model. In Fig. 4 we also compare the size of the Liver Transplantation Waiting List according to the official figures with the outcomes of the model.

**Table 2:** Summary of the results

Results	Current method	First method of [16]	Second method of [16]
Prevalence of Notified HCV Infections	163,902^a^ 169,382^b^	- 240,120^c^	- 227,074^c^
Prevalence of Non-Notified HCV in Brazil	1,433,638^a^ 1,446,771^b^	- 1,650,100^c^	- 1,632,300^c^
Total Prevalence of HCV in Brazil	1,597,540^a^ 1,616,153^b^	- 1,890,220^c^	- 1,859,374^c^

^a^Using only the official SINAN period (2000-2012) assuming zero notification incidence for all years and ages from 2000 backwards until 1932

^b^ Calculated from real data (2000-2012) and extending the data backwards assuming a sigmoidal decay until 1932

^c^ Taking the average number of cases reported annually to SINAN between 2004 and 2012, a period in which a steady state could be assumed

Amaku et al. [16] assumed a stationary situation so time dependence was removed from the equations. A system of differential equations was used to describe the densities with respect to age of susceptibles, reported individuals, non-reported individuals and recovered individuals. One parameter was the disease reporting rate *κ*. They used two methods.

In the first method it was assumed that the age-dependent force of infection *λ*(*a*) has a Gaussian shape with three scaling parameters. For a given value of *κ* the force of infection was used in the differential equations and was parametrically fitted to the age-dependent SINAN incidence data. The value of *κ* was then fitted heuristically to both the full age and time dependent SINAN data and the length of the LTWL. The fitted values of both *λ*(*a*) and *κ* were then used to find the total notified and non-notified HCV incidence data.

In the second method a different parametric function was fitted to the age-dependent SINAN incidence data. Given a value of *κ* they next used the differential equations to model the incidence. Again the value of *κ* was then fitted heuristically to both the full age and time dependent data and the length of the LTWL. The final fitted values of *κ* and the SINAN age-dependent incidence data were used to find the total notified and non-notified HCV incidence data.

The corresponding results, called the first method and second method in Table 2, were obtained using the following procedure. First, we assumed that the infection was in steady state from 2004 to 2012 and averaged the reported incidence. This reported incidence was extrapolated backwards until 1932. It is therefore not surprising that the published numbers in [16] including the third and fourth columns of Table 2 are larger than the figures obtained in this paper. The difference represents up to a certain point the state of the infection prior to 2000 and from this point of view the results seem to be consistent with what was believed about the infection in Brazil.

From the results of the current method expressed in Table 2 it is possible to observe that the difference between taking into account the constructed data backwards until 1932 and the official SINAN period of 2000-2012, reflects the significant contribution of this period to both the SINAN and the total prevalence of HCV in Brazil. Note that the artificially constructed incidence will manifest itself for individuals older than 40 years.

Figure 4 shows the comparison between the actual size of the LTWL as in Chaib et al. [17] and the result of the application of Eq. (17). The parameter *κ* was obtained in [16] by fitting the model to the LTWL. All other parameters were obtained independently of the LTWL. Figure 4 shows that using just this one fitted parameter the model accurately reproduces the whole LTWL time series. So we can assess the model as being reasonably accurate.

## Discussion

This paper is an attempt to provide a method to estimate the actual number of infected individuals (and other parameters related to transmission) of compulsory notifiable infectious diseases from the officially notified number of cases. Considering that, in the great majority of cases, the number of notified cases represents only a small but variable fraction of the total number of infected individuals, a reliable method of estimating the latter from the former can represent an important tool for public health policies. Notwithstanding the recognised importance of under-notification of most chronic infections, the tools to deal with this information gap proposed so far are varied and, to the best of our knowledge, there is currently no consensus about which is or are the most appropriate [3–8].

In a previous publication [16], a continuous time-dependent model for the estimation of the total number of HCV infected individuals in Brazil was proposed. In that paper, we assumed a steady state for the period between 2004 and 2012, and we concluded that the non-notified to notified ratio in the number of infections was about 7 to 1. The current work is an extension of that paper and we relaxed the steady state assumption. To do a calculation for individuals with age up to 80 years, we artificially extended the official notification database backwards from the year 2000 back to 1932. This artificially constructed database was intended only to illustrate the method. In addition, we discretised the variables time and age both because the notification database presents the number of cases per year and because the discrete model is easier to be implemented, both mathematically and computationally, than the continuous age and time corresponding model.

HCV is recently becoming virtually a 100%-curable disease due to antiviral treatments such as Ledipasvir/Acetonate/Sofosbuvir and others. So, there will be fewer and fewer individuals waiting for liver transplantation because of that. It is straightforward to modify the theoretical model to take account of this. If we have data on age, treatment and cure rates of individuals, let *ξ*(*a*, *t*) denote the rate at which notified infectious individuals of age *a* are given treatment and cured at time *t*. Then in the continuous model (2) in the first partial differential equation for *S*(*a,t*) there is an extra term

+*ξ*(*a*, *t*)*I* ^*N*^(*a*, *t*)

corresponding to infectious, notified, treated individuals who are cured and in the third partial differential equation of (2) for *I* ^*N*^(*a*, *t*) the term

$$ -\left(\mu \left(a,t\right)+{\alpha}^N\left(a,t\right)+{\gamma}^N\left(a,t\right)\right)\ {I}^N\left(a,t\right) $$

becomes

$$ -\left(\mu \left(a,t\right)+{\alpha}^N\left(a,t\right)+{\gamma}^N\left(a,t\right)+\xi \left(a,t\right)\right)\ {I}^N\left(a,t\right), $$

so *ϕ* ^*N*^(*a*, *t*) becomes

$$ \kern1.25em {\phi}^N\left(a,t\right)=\mu \left(a,t\right)+{\gamma}^N\left(a,t\right)+{\alpha}^N\left(a,t\right)+\xi \left(a,t\right). $$

Thus it is straightforward to model antiviral treatment.

The method presented in this paper is applicable to any compulsory notifiable infectious disease provided that one has information about at least two end-points of the natural history of the disease of interest, or carrying out an alternative diagnostic test in a representative sample of the affected population. For instance, for the case of HCV, we used the number of notified cases and the size of the Liver Transplantation Waiting List. For other diseases, in which one has only the number of notified cases, an alternative to the Liver Transplantation Waiting List depends on the disease one is interested in. For instance, for the case of dengue in a sufficiently small region, an age-dependent seroprevalence profile of a properly designed sample of the population would be sufficient. For infections like HIV, in addition to the reported number of cases, a sample representing each group of risk should be used.

The method demonstrated to be accurate in retrieving the number of infected individuals for the case of HCV as it fits the Liver Transplant Waiting List data (see Fig. 4) and the results are in good accordance with the previous estimations by Amaku et al. [16].

We have already said that the notification rate is the most important parameter in the model. This could be improved by various methods, for example public education about risk factors for HCV such as injecting drug use and new treatments, publicity campaigns, or screening programs, either of the general public or targeted high risk populations. Most important, however, would be a population-based seroprevalence study that could unequivocally determine individuals previously infected by HCV. The ratio of notified individuals to seropositive ones would determine the actual value of notification rate (*κ*).

In spite of its accuracy and simplicity, the method here presented has some important limitations that are worthwhile mentioning. Firstly, the model is data-greedy in the sense that a long time series of notified cases is necessary for the calculations. Secondly, the model has a large number of parameters whose values are not known with any precision for the great majority of cases. For example, as the model deals with long time series, demographic parameters such as the natural mortality rate are crucial for the calculations.

Notwithstanding those limitations, the model has the advantage that it can predict quantities that can be iteratively used to improve it. For instance, for HCV the model allows the calculation of the proportion of individuals that have the infection for τ years, that is the age of infection. If this can be checked from information from patients (e.g., blood transfusion time), the model can be improved immediately. This is thoroughly explained in Amaku et al. [16].

## Conclusions

We can conclude that the model proposed in this paper can be useful for estimation of the actual magnitude of endemic states of infectious diseases, particularly for those where the number of notified cases is only the tip of the iceberg. In addition, the method can be applied to other situations, such as the well-known under-reported incidence of criminality (for example rape), among others.

## Appendix

In this Appendix, we deduce the Eq. (7) from the main text. Let us define the function *I* ^*NN*^(*a* + *x*, *t* + *x*), which is a function that expresses the evolution of a cohort. Then

A1 $$ {\displaystyle \begin{array}{c}\frac{d}{dx}\left[{I}^{NN}\left(a+x,t+x\right)\right]={\lambda}_d\left(a+x,t+x\right)\ S\left(a+x,t+x\right)\\ {}-\left[{\kappa}_d\left(a+x,t+x\right)+{\phi}_d^{NN}\left(a+x,t+x\right)\right]{I}^{NN}\left(a+x,t+x\right),\end{array}} $$

where

$$ {\phi}_d^{NN}\left(a+x,t+x\right)={\mu}_d\left(a+x,t+x\right)+{\gamma}_d^{NN}\left(a+x,t+x\right)+{\alpha}_d^{NN}\left(a+x,t+x\right). $$

Multiplying both sides by $\exp \left[{\int}_0^x\left({\kappa}_d\left(a+z,t+z\right)+{\phi}_d^{NN}\left(a+z,t+z\right)\right) dz\right],$ we have

A2 $$ {\displaystyle \begin{array}{l}\frac{d}{dx}\left[\exp \left[{\int}_0^x\left({\kappa}_d\left(a+z,t+z\right)+{\phi}_d^{NN}\left(a+z,t+z\right)\right) dz\right]{I}^{NN}\left(a+x,t+x\right)\right]=\\ {}{\lambda}_d\left(a+x,t+x\right)\ S\left(a+x,t+x\right)\ \exp \left[{\int}_0^x\left({\kappa}_d\left(a+z,t+z\right)+{\phi}_d^{NN}\left(a+z,t+z\right)\right) dz\right].\end{array}} $$

So integrating we deduce that

A3 $$ {I}^{NN}\left(a,t\right)={I}^{NN}\left(a-1,t-1\right) $$

$$ {\displaystyle \begin{array}{l}\exp \left[-{\int}_0^1\left\{{\kappa}_d\left(a-1+z,t-1+z\right)+{\phi}_d^{NN}\left(a-1+z,t-1+z\right)\right\} dz\right]\\ {}+{\int}_0^1{\lambda}_d\left(a-1+x,t-1+x\right)S\left(a-1+x,t-1+x\right)\\ {}\ \exp \left[-{\int}_x^1\left\{{\kappa}_d\left(a-1+z,t-1+z\right)+{\phi}_d^{NN}\left(a-1+z,t-1+z\right)\right\} dz\right] dx.\end{array}} $$

The first term corresponds to non-notified individuals ages *a*-1 at time *t*-1 who remain infectious and non-notified at time *t* (when their age is *a*). The second term which we denote

$$ INCIDENCE\Big(a,\left(t-1,t\Big]\right) $$

is the density with respect to age *a* of the incidence of HCV in the cohort of individuals born at time *t*-*a* which occurs in the time interval (*t*-1,*t*] and is still infectious and not notified at time *t*.

Now, *I* ^*NN**^{*A*,*i*}, the absolute number of infectious non-notified individuals of age in the interval [*A*,*A* + 1) at time *t*_*i*_,

A4 $$ ={\int}_A^{A+1}{I}^{NN}\left(a,{t}_i\right) da, $$

A5 $$ \approx {I}^{NN}\left(A+\frac{1}{2},{t}_i\right), $$

taking the midpoint as an approximation.

Now from (A3) and (A4)

A6 $$ {\displaystyle \begin{array}{l}{I}^{NN\ast}\left\{A,i\right\}={\int}_A^{A+1}{I}^{NN}\left(a-1,{t}_i-1\right)\\ {}\kern1.50em \exp \left[-{\int}_0^1\left\{{\kappa}_d\left(a-1+z,{t}_i-1+z\right)+{\phi}_d^{NN}\left(a-1+z,{t}_i-1+z\right)\right\} dz\right] da\\ {}\begin{array}{llllll}& & & & & \end{array}+{\int}_A^{A+1} INCIDENCE\Big(a,\left({t}_i-1,{t}_i\Big]\right) da,\end{array}} $$

where for *a* ≤ 0, *I* ^*NN*^(*a*, *t*) is interpreted as zero. The last term in (A6), which we shall denote *INC*{*A*,*i*}, represents the incidence between times *t*_*i*_-1 and *t*_*i*_ of HCV that is still infectious and not notified at time *t*_*i*_, in the cohort born between times *t*_*i*_ *-A*-1 and *t*_*i*_-*A*. In the first term in (A6) again for the *a*-integration we take *a* = *A*+$\frac{1}{2}$ as an approximation, as the integration interval has length one.

$$ {\displaystyle \begin{array}{l}{I}^{NN\ast}\left\{A,i\right\}\approx {I}^{NN}\left(A-\frac{1}{2},{t}_i-1\right)\\ {}\begin{array}{lllll}& & & & \end{array}\exp \left[-{\int}_0^1\left\{{\kappa}_d\left(A-\frac{1}{2}+z,{t}_i-1+z\right)+{\phi}_d^{NN}\left(A-\frac{1}{2}+z,{t}_i-1+z\right)\right\} dz\right]\\ {}\begin{array}{lllllllllllllllllll}& & & & & & & & & & & & & & & & & & \end{array}+ INC\left\{A,i\right\}.\\ {}\begin{array}{cccc}& & & \end{array}={I}^{NN}\left(A-\frac{1}{2},{t}_i-1\right)\\ {}\begin{array}{lllll}& & & & \end{array}\ \exp \left[-{\int}_0^1\left\{{\kappa}_d\left(A-\frac{1}{2}+z,{t}_i\right)+{\phi}_d^{NN}\left(A-\frac{1}{2}+z,{t}_i\right)\right\} dz\right]+ INC\left\{A,i\right\},\end{array}} $$

as ${\kappa}_d\left(A-\frac{1}{2}+z,t\right)$ and ${\phi}_d^{NN}\left(A-\frac{1}{2}+z,t\right)$are the same for *t* ∈ (*t*_*i*_ − 1, *t*_*i*_].

$$ {\displaystyle \begin{array}{r}\approx {I}^{NN\ast}\left\{A-1,i-1\right\}\exp \left[-\frac{1}{2}\left({\kappa}_{A-1,i}+{\kappa}_{A,i}+{\phi^{NN}}_{A-1,i}+{\phi^{NN}}_{A,i}\right)\right]\\ {}+ INC\left\{A,i\right\},\end{array}} $$

because

(i) Noting that year *i*-1ends at time *t*_*i*_-1 we have
  $$ {I}^{NN}\left(A-\frac{1}{2},{t}_i-1\right)\approx {I}^{NN^{\ast }}\left\{A-1,i-1\right\}, $$ by (A5).

(ii) for $z\in \left[\left.0,\frac{1}{2}\right)\right.,$ ${\kappa}_d\left(A-\frac{1}{2}+z,{t}_i\right)={\kappa}_{A-1,i}$ and for $z\in \left[\left.\frac{1}{2},1\right]\right.,{\kappa}_d\left(A-\frac{1}{2}+z,{t}_i\right)={\kappa}_{A,i}$.

## Abbreviations

CND: Compulsory notifiable disease
HCV: Hepatitis C virus
HIV: human immunodeficiency virus
LTWL: Liver Transplantation Waiting List
SINAN: Sistema de Informação de Agravos de Notificação (National Information System of Notifiable Diseases)
SIR: Susceptible-Infected-Removed
WHO: World Health Organization
